# Evaluation of Clinical and Radiographic Parameters as Dental Indicators for Postmenopausal Osteoporosis

**DOI:** 10.3290/j.ohpd.a44688

**Published:** 2020-07-04

**Authors:** Tanveer Alam, Ibrahim AlShahrani, Khalil Ibrahim Assiri, Salem Almoammar, Rafi A Togoo, Mannakandath Luqman

**Affiliations:** a Assistant Professor, Department of Diagnostic Science and Oral Biology, King Khalid University College of Dentistry, Abha, Saudi Arabia. Idea, clinical examination of subjects, proofread the paper.; b Associate Professor, Department of Pediatric Dentistry and Orthodontics, King Khalid University College of Dentistry, Abha, Saudi Arabia. Literature review, proofread the paper.; c Assistant Professor, Department of Diagnostic Science and Oral Biology, King Khalid University College of Dentistry, Abha, Saudi Arabia. Study design, proofread the paper.; d Assistant Professor, Department of Pediatric Dentistry and Orthodontics, King Khalid University College of Dentistry, Abha, Saudi Arabia. Manuscript writing and final revision.; e Professor, Department of Pediatric Dentistry and Orthodontics, King Khalid University College of Dentistry, Abha, Saudi Arabia. Statistical analysis, manuscript revision and proofreading.; f Assistant Professor, Department of Diagnostic Science and Oral Biology, King Khalid University College of Dentistry, Abha, Saudi Arabia. Data collection and interpretation, proofread the paper.

**Keywords:** bone mineral density, dual-energy X-ray absorptiometry, osteoporosis, panoramic radiography, postmenopausal women

## Abstract

**Purpose:**

In the early stages, osteoporosis is relatively a silent disease characterised by low bone density with microarchitectural deterioration of the bone tissue leading to enhanced bone fragility. The objective of this study was to evaluate the relationship between age, body mass index, oral signs, and osteoporosis among postmenopausal women.

**Materials and Methods:**

The study included postmenopausal women who were divided into two groups of subjects. The osteoporotic group comprised 30 patients with osteoporosis who were diagnosed using dual-energy X-ray absorptiometry, and the non-osteoporotic group (control group) comprised 30 subjects with no evidence of osteoporosis. Panoramic radiography was performed, followed by the acquisition of two direct digital intraoral periapical radiographs from the mandibular premolar-molar region.

**Results:**

Chi-square test revealed a statistically significant difference (p = 0.001) in the mandibular cortical shape index between the two groups. However, a statistically non-significant difference in cortical width, the panoramic mandibular index, mandibular alveolar bone resorption degree, fractal dimension, and mean number of teeth was found between the two groups. A statistically significant difference was observed in the mean age between the osteoporotic and non-osteoporotic groups.

**Conclusion:**

The mandibular cortical index findings (MCI) on panoramic radiograph are effective indicators of osseous changes in postmenopausal osteoporosis, thereby determining early prediction of osteoporotic fracture risk and reducing its related morbidity.

The word ‘osteoporosis’ means porous bone. The loss of bone mineral density (BMD) associated with osteoporosis is the result of disease progression. Osteoporosis is usually accompanied by severe pain, muscle spasms, weakened bones and pathologic fractures. Almost 10% of the world’s population and 30% of postmenopausal women have osteoporosis.^1^ A previous study suggested that 20% of women aged over 50 years in India had osteoporosis.^13^

The gold standard for diagnosing osteoporosis is BMD, which is measured using dual-energy X-ray absorptiometry (DEXA). In 1960, a study first suggested an association between osteoporosis and oral bone loss.^4^ Since the teeth are embedded in the jaws, osteoporotic changes can cause tooth loss or erosion of the jaw bones. Researchers have considered dental radiographs as a screening tool as they are used in routine dental screening and can help the dentist evaluate the whole dentition and jaw bones. The aim of the present study was to evaluate various parameters for use as indicators of postmenopausal osteoporosis.

The objectives of the present study were to evaluate the relationship between age, body mass index (BMI), oral signs and osteoporosis. The oral signs, which included cortical width (CW), the panoramic mandibular index (PMI), mandibular alveolar bone resorption degree (M/M ratio), mandibular cortical shape index (MCI), and the number of mandibular teeth, were evaluated using panoramic radiographs, whereas fractal dimension (FD) analysis was used to evaluate the mandibular trabecular bone by using direct digital intraoral periapical radiographs.

We also aimed to determine whether radiographic changes could be detected in the mandible of patients with postmenopausal osteoporosis and whether these changes could be used as a diagnostic tool to differentiate normal subjects from patients with osteoporosis. Finally, we analysed the usefulness of these parameters as indicators of postmenopausal osteoporosis.

## Materials and Methods

An analytical study was conducted at the Department of Diagnostic Science and Oral Biology. Before commencing the study, ethical approval was obtained from the Scientific Research Committee, King Khalid University, College of Dentistry, Abha.

Our study included 60 postmenopausal women who were enrolled from the outpatient department of Oral Medicine and Radiology. The subjects were divided into two groups: the osteoporotic group comprised 30 women diagnosed with osteoporosis by using DEXA, and the non-osteoporotic group (control group) comprised 30 women with no evidence of osteoporosis. The study included women aged 45–60 years with/without osteoporosis in a state of natural menopause (ie, ceased menstruation for at least 1 year), willing to participate in the study, and free from any systemic disease that would affect bone metabolism. Patients on any specific medication or hormones, which were known to have adverse effects on bone metabolism, smokers, alcoholics and patients with osteopenia were excluded from the study.

### Measurement of BMD

DEXA of the lumbar spine was performed using the Lunar DPX-IQ bone densitometer (GE Medical systems, Madison, WI, USA), which uses an X-ray tube with a switched pulsed dual energy of 140 and 100 kVp at a frequency of 50 Hz with a scan speed of 0.321 mm/s. ‘T’ score is an expression of BMD values in terms of the standard deviations from the normal mean value of a young female adult. The World Health Organization (WHO) classifies the patients as normal (T-score > –1.0), osteopenic (T-score –1.0 to –2.5), or osteoporotic (T-score < –2.5) on the basis of the BMD data of the lumbar spine. The data regarding menopausal status, age, weight and height were recorded during DEXA measurement.

### Radiographic Measurements

Panoramic radiography (OP-200, Finland) was performed on all the subjects and then followed by the acquisition of two digital intraoral periapical radiographs (Focus, Finland) from the mandibular premolar-molar region. The panoramic radiographs were acquired at 76 kV and 10 mA for 12 s. The digital radiographs were acquired at 65 kV, 10 mA and 2.5 mm equivalent total filtration. Image J (1.28) software was used for image processing and analysis.

**Fig 1:**
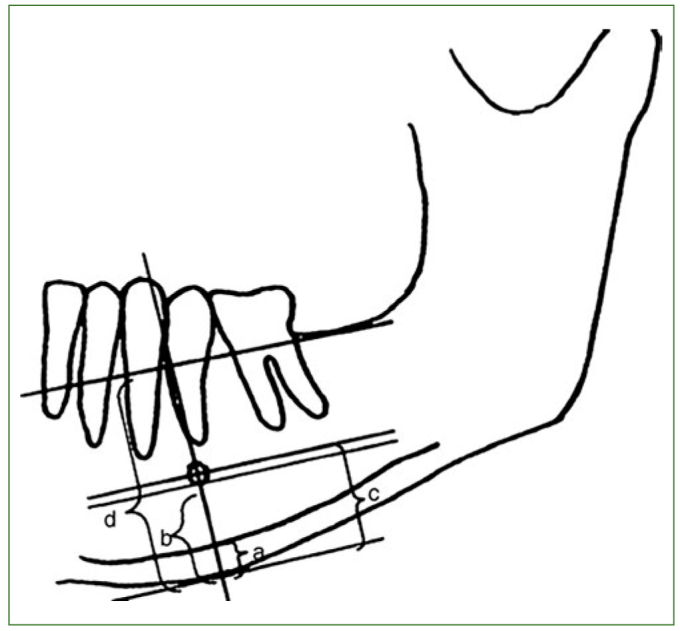
Measurements on the panoramic radiograph. a = cortical width (CW); a/b = panoramic mandibular index (PMI); d/c = mandibular alveolar bone resorption index (MM ratio).

The indices (eg, mandibular CW, PMI, and M/M ratio) were measured by drawing lines parallel to the long axis of the mandible and tangential to the inferior border of the mandible. Further, a line perpendicular to this tangent intersecting the inferior margin of the mental foramen was drawn and measurements were taken along this perpendicular line, as shown in Figure 1. Mandibular CW is the thickness of the mandibular cortex on this line (a). It was measured on the right and left sides, and the mean value was taken as the CW of the patient. The PMI is the ratio of the thickness of the mandibular cortex to the distance between the mental foramen and inferior mandibular cortex. Mandibular height was divided by the height from the centre of the mental foramen to the inferior border of the mandible to measure the M/M ratio (Fig 1).

The MCI is related to mandibular BMD and serves as a valuable indicator of mandibular porosity.^10,21^ The MCI on the panoramic radiograph was determined by observing the mandible distally from the mental foramen bilaterally and was categorised using Klemetti’s classification,^10^ which is as follows: C1, the endosteal margin of the cortex is even and sharp on both sides; C2, the endosteal margin shows semilunar defects (lacunar resorption) and/or seems to form endosteal cortical residues on one or both sides; and C3, the cortical layer forms heavy endosteal cortical residues and is clearly porous.

**Fig 2:**
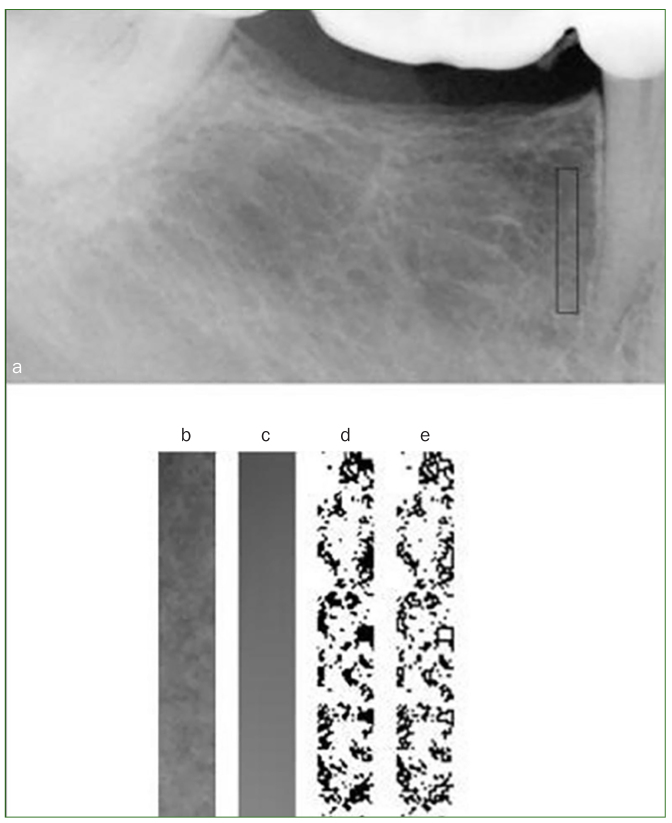
The original radiograph with a region of interest (ROI). (a) The selected ROI. (b) The result of blurring this region. (c) The result of subtracting B from A and adding 128. (d) Inverted binary version of the image. (e) Outlines of the trabeculae are obtained.

FD analysis is a quantitative method to measure complex geometric structures within the image and is used to predict osteoporosis. An increase in the FD number indicates an increasing complexity of the exhibited patterns in an image. FD analysis was done as follows. First, the 16-bit direct digital radiographs were converted to 8-bit images. Rectangular regions of interest with identical dimensions (23 × 155 pixels) for all radiographs were created between the second premolar and first molar regions. Digital images were segmented to binary images by using a technique described by White and Rudolph, by blurring using a Gaussian filter, image subtraction, conversion into a binary image, and inversion to highlight the trabecular outlines for better description^21^ (Fig 2). The mean values of both the right and left sides of the mandible in the premolar-molar region were used to calculate the FD.

### Statistical Analysis

The collected data from all the groups were imported to SPSS for Windows, Version 16.0 (SPSS, Chicago, IL, USA). Since the distribution met the assumptions of normality, the Mann–Whitney U test and chi-square test were used to analyse the data. The statistical significance level was set at p < 0.05.

## Results

Women aged 45–60 years were included in the study. The Mann–Whitney U test showed a statistically significant difference (p = 0.000) in the mean age between the osteoporotic (56.5 years) and non-osteoporotic (50.8 years) groups. A statistically non-significant difference (p = 0.164) in the mean number of mandibular teeth was seen between the osteoporotic (11.7) and non-osteoporotic (12.6) groups. The Mann–Whitney U test showed a statistically non-significant difference in the BMI between the two groups. A statistically non-significant difference was also found for CW between the osteoporotic and non-osteoporotic groups (Table 1). A statistically non-significant difference in the PMI was found between the two groups (Table 1). A statistically non-significant difference was also observed in the M/M ratio (Table 1) and for the FD (Table 1) between the two groups. The chi-square test revealed a statistically significant difference in the MCI between the two groups (Table 2). The risk of osteoporosis in the C3 category was 11.36 times higher than that in the C2 category and 10.64 times higher for C2 than for C1; moreover, the risk of osteoporosis in the C3 category was 111.11 times higher than that in the C1 category (Table 3).

**Table 1:** Comparison of cortical width, the panoramic mandibular index, mandibular alveolar bone resorption degree (M/M ratio), and fractal dimension between the osteoporotic and non-osteoporotic groups

Cortical width		Panoramic mandibular index		M/M ratio		Fractal dimension	
Osteoporotic (n = 30)	Non-osteoporotic (n = 30)	Osteoporotic (n = 30)	Non-osteoporotic (n = 30)	Osteoporotic (n = 30)	Non-osteoporotic (n = 30)	Osteoporotic (n = 30)	Non-osteoporotic (n = 30)
4.9 (± 1.0)	4.8 (± 0.9)	0.36 (± 0.08)	0.35 (± 0.08)	1.98 (± 0.24)	2.05 (± 0.29)	1.65 (± 0.02)	1.64 (± 0.02)
P value 0.876#		P value 0.605#		P value 0.515#		P value 0.326#	

# Statistically non-significant.

**Table 2:** Cortical index of the osteoporotic and non-osteoporotic groups

Parameter	Osteoporotic (n = 30)		Non-osteoporotic (n = 30)		P value
Cortical index	Mean	SD	Mean	SD	
Grade I	5	16.7	18	60.0	0.001*
Grade II	20	66.7	12	40.0	
Grade III	5	16.7	0	0	

*Statistically significant. SD, standard deviation.

**Table 3:** Univariate analysis of cortical index classifications

Odds ratio for osteoporosis (present-absent)	OR value	95% confidence interval	
		Lower	Upper
C1 to C2	0.094	0.014	0.478
C2 to C3	0.088	0.010	0.790
C1 to C3	0.009	0.001	0.088

C, cortical shape index; OR, odds ratio.

Binary logistic regression analysis was used to analyse the age to generate an equation for the probability of a new subject developing osteoporosis. The probability value (Pv) was more than 0.50 in women aged more than 61 years. If the Pv exceeded 0.50, then the subject was likely to develop osteoporosis. The overall accuracy of the prediction of subjects developing osteoporosis was 95.87%.

## Discussion

According to the WHO, osteoporosis is the second biggest global healthcare problem. Postmenopausal women constitute more than 15% of the population with osteoporosis in developed countries, whereas this rate is 5–8% in the less developed regions of the world.^22^ By 2030, the world population of menopausal and postmenopausal women is expected to increase to 1.2 billion, with 47 million new women added each year.^6^ Osteoporosis is more prevalent in women than in men because of oestrogen deficiency in postmenopausal women and calcium loss during pregnancy and menstruation. Riggs and Melton (1986) reported that postmenopausal osteoporosis characteristically affects women within 15–20 years after menopause.^17^

Skeletal BMD is evaluated using DEXA of the spine, and it serves as a gold standard for diagnosing patients with osteoporosis. Horner et al (1996) used DEXA both in the mandible and in the forearm and found a good correlation. However, the DEXA technique in the mandible is difficult and expensive and is more suitable for use in edentulous patients.^8^

Age is a useful clinical predictor and has often been reported as a risk factor for osteoporosis. Our findings were consistent with those of Nackaerts et al (2008) and Yasar and Akgunlu (2006) who reported that osteoporosis is a major health problem at the time of menopause in women aged 50–65 years.^15,23^

The protective effects of high body weight on bone density and fracture risk are attributed to the stimulation of bone formation by greater mechanical loading, conversion of adrenal androgens to oestrogens in fat, and the shock-absorbing properties of subcutaneous fat. In this study, no statistical difference was observed in the BMI between the two groups, which was in accordance with the findings of Yasar and Akgunlu (2006).^23^

Most studies on the relationship between jaw bone density and other skeletal sites have been performed in the mandible, on the basis of the concept that the basal area of the mandible, in the region of the mental foramen, is an area of the jaw that roughly fulfils the demands of a standard site. Halling et al (2005) suggested that the anterior maxilla is a sensitive site for distinguishing patients with osteoporosis from normal subjects because of the relatively large amount of trabecular bone at this site.^5^ Von Wowern and Stoltze (1979) demonstrated using bone morphometry that the buccal cortical bone mass of the mandible was significantly correlated with the metacarpal index.^19^

In this study, the mental index was chosen to allow for the quantification of mandibular bone mass, which could be easily measured using standardised intraoral radiography. Horner and Devlin (1998) stated that the cortex measured on the panoramic radiograph below the mental foramen is a true cortical bone, whereas that measured at the gonial angle is a projection of the bony ridge related to the insertion of the masseter and medial pterygoid muscles.^9^

In our study, mandibular CW showed no statistical significance in the osteoporotic and non-osteoporotic groups. This was consistent with the findings of Mohajery et al (1992) and Kribbs et al (1989). Thus, the cortical thickness of the mandible on the panoramic radiograph does not indicate osteoporosis.^12,14^

We found that the PMI was not statistically significant between the two groups. Our observations were in accordance to those of studies by Watson et al (1995) and Drozdzowska et al (2002).^2,20^ Finding a positive correlation between the PMI and osteoporosis in a population of postmenopausal middle-aged women is difficult.

We also found that the M/M ratio showed no difference between the two groups. The results were similar to those of Hirai et al (1993) who indicated the association of the M/M ratio with the severity of osteoporosis in elderly edentulous men and women.^7^

In this study, a significantly higher proportion of patients with osteoporosis had a higher MCI than did those without osteoporosis. Nakamoto et al (2003) reported that it is likely that general dental practitioners tend to select women with a C3 cortex rather than a C1 or C2 cortex on panoramic radiographs in their clinical practice when identifying women with a low BMD, because it is easier for them to diagnose the C3 cortex.^16^ Mandibular cortical erosion suggests bone turnover in the form of increased bone resorption in postmenopausal women.

The mean number of teeth present in subjects with and without osteoporosis in our study was not statistically significant. Taguchi et al (2004) suggested that the number of teeth present is a useful predictive indicator of vertebral fracture due to osteoporosis.^18^ Krall et al (1994) suggested that systemic bone loss might contribute to tooth loss in healthy postmenopausal women.^11^ However, Klemetti et al (1994) found no correlation between the number of teeth present and BMD in postmenopausal women because vertebral fragility depends on cortical BMD.^10^

Bone remodelling is more extensive in the trabecular bone than in the compact bone, and thus osteoporosis is more extensive in the trabecular bone. Geraets and Van der Stelt (2000) stated that most reports based on radiographs in vivo found an association between osteoporosis and increased values of FD.^3^

## Conclusion

Osteoporosis, especially in postmenopausal period of women, causes a reduction in bone mass and density significantly impairing the quality of life. This study concludes that the MCI findings on panoramic radiograph are effective indicators of osseous changes in postmenopausal osteoporosis, thereby determining early prediction of osteoporotic fracture risk and reducing its related morbidity. These findings significantly highlight the importance of using routine panoramic radiographs as a diagnostic tool for the detection of osteoporosis. Our findings strongly suggest the importance of age as a risk factor for osteoporosis along with MCI, which serves as a potential screening tool for osteoporosis and referral for bone densitometry tests. In addition, parameters such as CW, PMI, M/M ratio, FD, and number of mandibular teeth did not necessarily reflect the presence or absence of osteoporosis. This requires further longitudinal studies to establish the cause and effect relationships between the risk factors and the outcome measures for osteoporosis.

### Recommendations

The main goal of osteoporosis intervention lies in preventing and/or slowing the extent of bone loss. More community awareness programmes aimed at early detection of osteoporosis are necessary to help patients in ensuring that corrective measures are initiated at early stages of the disease to prevent morbidity. It is recommended that women should be educated in the importance of having a balanced diet with the necessary amounts of minerals and vitamins – especially calcium and vitamin D – so that they maintain bone density, and hence lead a healthy life free from bone disorders such as osteoporosis.
